# Predictive value of antioxidant and thyroid function indicators for non-suicidal self-injury in adolescents with major depressive disorder

**DOI:** 10.3389/fpsyt.2026.1831669

**Published:** 2026-06-01

**Authors:** Kefei Yang, Lewei Liu, Xianlong Zhang, Xianlin Sun, Pei Tang, Kai Zhang, Huanzhong Liu

**Affiliations:** 1 Department of Psychiatry, The Fourth Affiliated Hospital of Anhui Medical University, Hefei, Anhui, China; 2 Department of Psychiatry, School of Mental Health and Psychological Sciences, Anhui Medical University, Hefei, Anhui, China

**Keywords:** adolescent, indirect bilirubin, major depressive disorder, non-suicidal self-injury, thyroid-stimulating hormone

## Abstract

**Background:**

Non-suicidal self-injury (NSSI) is highly prevalent in adolescents with major depressive disorder (MDD); however, its underlying pathophysiological mechanisms remain incompletely elucidated. Emerging evidence suggests a potential association between antioxidant markers, thyroid function parameters, and the occurrence of NSSI, although research in this domain remains limited. Accordingly, this study aimed to investigate the predictive efficacy of combining antioxidant and thyroid biomarkers with clinical symptoms for NSSI in adolescents with MDD.

**Methods:**

This study recruited 162 adolescents with MDD between September 2022 and January 2026. Participants were stratified into groups based on the presence or absence of NSSI, in accordance with DSM-5 diagnostic criteria. Multidimensional scales were employed to assess the severity of depression, anxiety, perceived stress, and internet addiction (IA). Concurrently, blood samples were collected to measure bilirubin levels and thyroid function parameters. Stepwise logistic regression analysis was subsequently performed to identify independent risk factors associated with NSSI. Finally, receiver operating characteristic (ROC) curves were constructed to quantify the predictive performance of these identified independent factors.

**Results:**

The prevalence of NSSI in adolescents with MDD was 57.4%. Multivariate logistic regression analysis identified females (OR = 2.246, 95% CI = 1.032-4.888, *P* = 0.041), HAMD-17 score (OR = 1.183, 95% CI = 1.088-1.286, *P* < 0.001), indirect bilirubin (OR = 0.890, 95% CI = 0.797-0.995, *P* = 0.040), and TSH (OR = 2.060, 95% CI = 1.254-3.385, *P* = 0.004) as independent predictors of NSSI. Furthermore, ROC curve analysis further demonstrated that the four-item combination of sex, HAMD-17 score, indirect bilirubin, and TSH (AUC = 0.776, 95% CI = 0.701-0.850, *P* < 0.001) had a better ability to identify NSSI.

**Conclusion:**

Adolescents with MDD, particularly females, represent a high-risk population for NSSI. Reduced levels of indirect bilirubin coupled with elevated TSH levels may constitute the underlying pathophysiological basis of NSSI and demonstrate significant clinical predictive value. In the future, targeted intervention strategies focusing on the antioxidant defense system and thyroid function may offer novel therapeutic avenues for the management of NSSI.

## 1 Introduction

Major Depressive Disorder (MDD) has emerged as a leading contributor to the global disease burden in adolescents (1). Among the myriad clinical manifestations, non-suicidal self-injury (NSSI) warrants particular attention. NSSI is defined as the deliberate, direct destruction of one’s own body tissue (e.g., cutting, burning, or hitting) without suicidal intent, and in a manner not socially sanctioned (2). Epidemiological data indicate that the overall prevalence of NSSI in non-clinical adolescent populations is approximately 22.0% (3); however, this rate escalates significantly in adolescents with MDD, exceeding 40% (4). More critically, NSSI not only severely impairs academic performance, interpersonal relationships, and social functioning but also serves as a key predictor of future suicide attempts (2, 5). Consequently, there is an urgent clinical imperative to comprehensively investigate the multidimensional risk factors underlying NSSI behaviors in adolescents with MDD and to construct predictive models. Such efforts are essential for the early identification of high-risk individuals and the formulation of personalized intervention strategies.

Existing research indicates that a spectrum of clinical symptoms is closely intertwined with the onset and progression of NSSI. Regarding affective symptoms, depression and anxiety not only constitute the core pathological features of MDD but also serve as critical psychodynamic mechanisms that precipitate and sustain NSSI behaviors. A study based on network analysis further revealed a complex interactive network between NSSI and symptoms of depression and anxiety in adolescents, wherein depressive symptoms exhibited greater centrality (6). In terms of stress response, perceived stress levels represent another pivotal risk factor. Elevated chronic stress not only activates the hypothalamic-pituitary-adrenal (HPA) axis, leading to dysregulated cortisol secretion rhythms, but also significantly impairs prefrontal control over impulsive behaviors, thereby increasing susceptibility to NSSI (7–9). Beyond these internalizing symptoms, behavioral and environmental factors cannot be overlooked. Among them, internet addiction (IA), as a significant comorbid condition, is increasingly exacerbating the risk of NSSI (10). Excessive internet use not only aggravates social anxiety and depressive symptoms but also exposes individuals to cyberbullying, which acts as an independent predictor of NSSI (11, 12). A meta-analysis encompassing 41 studies identified a moderate positive correlation between IA and NSSI in adolescents (13). Consequently, in clinical assessment and intervention, it is imperative to recognize the pivotal role played by psychopathological symptoms in the etiology of NSSI.

Accumulating evidence indicates that the pathogenesis of MDD is closely linked to an imbalance within the body’s oxidative-antioxidant systems (14, 15). Excessive accumulation of reactive oxygen species (ROS) can lead to lipid peroxidation, protein denaturation, and DNA damage, subsequently triggering neuroinflammation and neuronal apoptosis, particularly affecting brain regions involved in emotion regulation, such as the prefrontal cortex and hippocampus (16). As the end product of heme metabolism, bilirubin is one of the most potent endogenous antioxidants in the human body, effectively scavenging free radicals and inhibiting inflammatory responses (17). Preliminary observations suggest that depressed adolescents exhibiting NSSI behaviors often present with more severe oxidative stress states and lower serum bilirubin levels (18). Furthermore, uric acid represents another critical antioxidant marker. A meta-analysis demonstrated that uric acid levels are significantly lower in patients with MDD compared to healthy controls (19). Wu et al., through an investigation of adolescents with mood disorders, further identified that elevated serum uric acid levels act as a protective factor against self-harm behaviors (20). Nevertheless, systematic research regarding the specific predictive value of antioxidant markers, such as bilirubin and uric acid, for NSSI in adolescents remains scarce. Whether these biomarkers can be combined with clinical symptoms to optimize the predictive efficacy for NSSI warrants further validation.

Concurrently, the robust association between thyroid dysfunction and psychiatric disorders has been widely recognized (21). Thyroid hormones not only regulate basal metabolic rate but also serve as critical modulators of brain development, neurotransmitter synthesis, and synaptic plasticity (22). Subclinical thyroid abnormalities, characterized by fluctuations in thyroid-stimulating hormone (TSH) levels or alterations in thyroxine (T4), are prevalent in adolescents with MDD and are frequently associated with more severe clinical manifestations (23). Notably, emerging evidence suggests that aberrations in thyroid parameters, including TSH and T4, may be significantly correlated with an elevated risk of NSSI (24, 25). However, research on the ability of thyroid markers to differentiate MDD subtypes with and without NSSI in adolescents remains scarce.

Therefore, this study aimed to integrate multidimensional clinical features (depressive and anxiety symptoms, perceived stress, and IA with antioxidant profiles and thyroid function indicators. By systematically elucidating the associations between these factors and NSSI behaviors in adolescents with MDD, we sought to develop a comprehensive risk prediction model.

## 2 Methods

### 2.1 Participants

This study employed a convenience sampling strategy to recruit 162 adolescents with MDD from the inpatient psychiatric department of the Fourth Affiliated Hospital of Anhui Medical University between September 2022 and January 2026. Inclusion criteria were as follows: (1) Age ranging from 12 to 18 years. (2) Meeting the diagnostic criteria for MDD as outlined in the Diagnostic and Statistical Manual of Mental Disorders, Fifth Edition (DSM-5). Exclusion criteria included: (1) A current diagnosis of other psychiatric disorders (e.g., schizophrenia). (2) Presence of severe infections, organic brain lesions, or significant systemic medical conditions (e.g., respiratory diseases, thyroid dysfunction). (3) Inability to cooperate with the study procedures or failure of legal guardians to provide valid informed consent. The study was conducted in strict adherence to the principles of the Declaration of Helsinki and received approval from the Ethics Committee of the Fourth Affiliated Hospital of Anhui Medical University (Approval No. 202009-KYXM-04). Written informed consent was obtained from all participating adolescents and their legal guardians following a comprehensive explanation of the study procedures. Given that the study population comprised adolescents with NSSI, a vulnerable group, rigorous risk management protocols were implemented. All assessments were conducted in coordination with and under the supervision of the participants’ attending psychiatrists to ensure that the research procedures did not interfere with routine clinical care. Furthermore, an immediate feedback mechanism was established: any significant findings indicating acute risk (e.g., escalated suicidal ideation or severe urges for self-harm) identified during the assessment were promptly communicated to the primary treating physician. This allowed for the timely initiation of necessary crisis intervention measures, thereby ensuring the safety and well-being of the participants.

### 2.2 Measuring instruments

#### 2.2.1 General data

In this study, a self-developed questionnaire was employed to collect participants’ demographic and clinical characteristics, including sex, age, body mass index (BMI), age at onset, duration of illness, and the specific classes of antidepressant medications currently prescribed.

#### 2.2.2 NSSI

The assessment of NSSI was conducted in accordance with the criteria outlined in the DSM-5. The evaluation focused on the following key domains: (1) Frequency and severity: The occurrence of self-injurious behavior intended to cause only mild to moderate physical damage on at least five days within the past year. (2) Purpose and mental state: The behavior is undertaken to regulate negative emotions or interpersonal states and is associated with either a preoccupation with self-injury prior to the act or difficulty resisting the urge to engage in the behavior. (3) Functional impairment: The behavior causes clinically significant distress or interference in important areas of functioning, such as interpersonal relationships or academic performance. (4) Exclusion criteria: The behavior is not socially sanctioned (e.g., body piercing or tattooing), does not occur exclusively during episodes of stereotyped behaviors associated with neurodevelopmental disorders, and is not attributable to another mental disorder, medical condition, or substance use.

#### 2.2.3 Depressive symptoms

Depression severity was quantified using the 17-item Hamilton Depression Rating Scale (HAMD-17) (26). This instrument comprises 17 items, yielding a total score ranging from 0 to 52, with higher scores indicating greater symptom severity. This scale has been widely used to assess the severity of depression in Chinese adolescent patients and has demonstrated good reliability and validity (27, 28).

#### 2.2.4 Anxiety symptoms

Anxiety levels were assessed using the Hamilton Anxiety Rating Scale (HAMA) (29). The scale consists of 14 items, each rated on a 5-point Likert scale, yielding a total score between 0 and 56. Higher scores indicate more severe anxiety symptoms. Currently, the Chinese version of the HAMA has been widely used in clinical studies involving Chinese adolescents (30, 31).

#### 2.2.5 Stress levels

Perceived stress levels were assessed using the Perceived Stress Scale (PSS) (32). This instrument is designed to evaluate the degree of stress individuals perceive in response to unpredictable, uncontrollable, or overwhelming life events. The scale comprises 10 items, each rated on a 5-point Likert scale, yielding a total score between 0 and 40. Higher scores indicate greater levels of perceived stress. The PSS has been validated for assessing perceived stress among Chinese adolescents (33).

#### 2.2.6 IA

IA was assessed using the Internet Addiction Test (IAT) (34). The scale comprises 20 items addressing the frequency and contexts of internet use. Each item is scored on a 5-point Likert scale, yielding a total score between 20 and 100, with higher scores indicating more severe IA. The Chinese version of the IAT has been extensively validated among adolescent populations in China and demonstrates robust applicability (35).

### 2.3 Measurement of biochemical parameters

All participants underwent an overnight fast of at least 8 hours, with fasting venous blood samples collected between 06:00 and 07:00. Samples were immediately transported to the Department of Clinical Laboratory at the Fourth Affiliated Hospital of Anhui Medical University for centrifugation and analysis. Serum levels of total bilirubin, direct and indirect bilirubin, and uric acid were measured using a Sysmex XN-9000 automated hematology analyzer. Additionally, concentrations of triiodothyronine (T3), T4, and TSH were determined via a Roche Cobas 8000 e801 fully automated chemiluminescence immunoassay system. The normal reference ranges for total bilirubin, direct bilirubin, indirect bilirubin, uric acid, T3, T4, and TSH are 5-21 μmol/L, 0-5.1 μmol/L, 0-17 μmol/L, 208-428 μmol/L, 0.35-1.93 ng/mL, 48.7-117.20 μg/L, and 0.35-4.94 μIU/mL, respectively.

### 2.4 Quality control

To ensure the reliability of disease diagnosis and NSSI assessment, all enrolled patients underwent the Structured Clinical Interview for DSM-5, Research Version (SCID-5-RV), conducted by two psychiatrists with attending physician or higher professional titles. Diagnoses were strictly made according to the criteria outlined in DSM-5. During data collection and scale assessments, two clinically trained evaluators who had passed rigorous consistency training and qualification tests independently performed the evaluations. The inter-rater reliability, measured by intraclass correlation coefficients (ICCs), exceeded 0.80 for all assessors, ensuring scoring consistency. Furthermore, all enrolled patients were in the acute phase of MDD, and their clinical assessments and blood sample collections were completed on the same day to minimize the impact of temporal variability on the results.

### 2.5 Statistical analysis

Statistical analyses were performed using IBM SPSS version 23.0. For continuous variables, normality was first assessed using the Kolmogorov-Smirnov (K-S) test. Variables conforming to a normal distribution were presented as mean ± standard deviation (SD), with between-group comparisons performed using independent-samples t-tests. Non-normally distributed variables were expressed as median (interquartile range) [M (P_25_, P_75_)], and group differences were evaluated using the Mann-Whitney U test. Categorical variables are summarized as frequencies and proportions [n (%)], with intergroup comparisons conducted using the chi-square (χ^2^) test. To identify independent predictors of NSSI in adolescents with MDD, a binary logistic regression model was constructed. Variable selection followed a “Forward: Likelihood Ratio” stepwise strategy, with the initial candidate pool comprising variables showing an association in univariate analysis (*P* < 0.10). Multicollinearity diagnostics revealed that variance inflation factor (VIF) values for all independent variables were below 5.0, indicating no significant multicollinearity within the model. Finally, the predictive value of statistically significant independent factors was validated using receiver operating characteristic (ROC) curve analysis, including calculations of the area under the curve (AUC), sensitivity, and specificity. All statistical tests were two-tailed, and a *P*-value < 0.05 was considered statistically significant.

## 3 Results

### 3.1 Comparison of clinical characteristics between NSSI and non-NSSI groups

**Figure 1:**
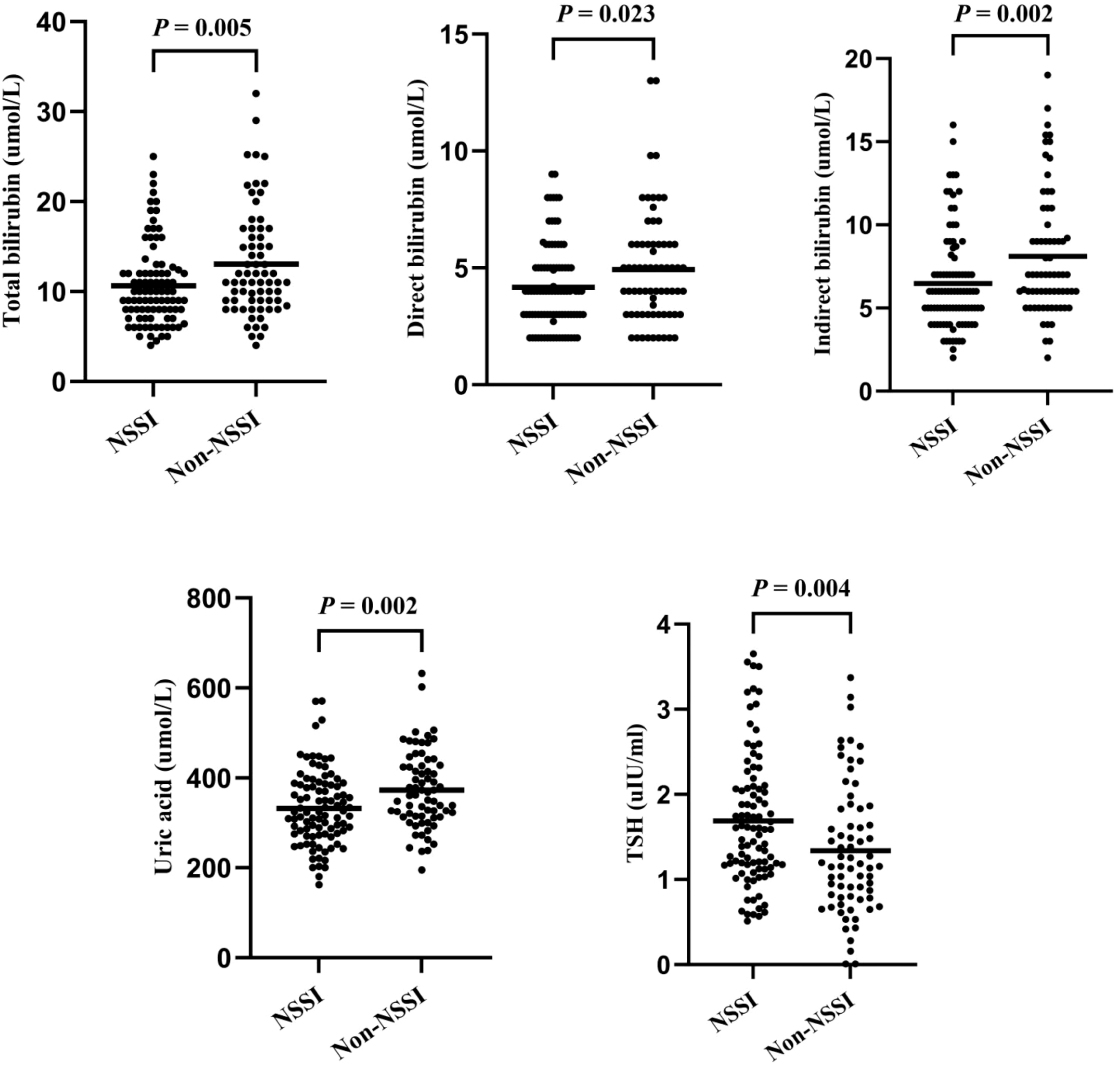
Comparison of hematological parameters between NSSI and non-NSSI groups.

The study comprised 162 adolescents with MDD, of whom 57 were male (35.2%). The mean age of the participants was 15.26 ± 1.63 years. The mean age at onset was 13.71 ± 1.94 years, with a mean illness duration of 18.33 ± 15.66 months. Notably, the prevalence of NSSI within this sample was 57.4%. Compared with the non-NSSI group, patients in the NSSI group exhibited a significantly higher proportion of females, as well as younger current age and earlier age at onset. Regarding psychological assessments, the NSSI group demonstrated significantly elevated scores on the HAMD-17, HAMA, PSS, and IAT. Biochemical analyses revealed that levels of total bilirubin, direct bilirubin, indirect bilirubin, and uric acid were significantly lower in the NSSI group, whereas TSH levels were significantly higher (all *P* < 0.05) (**Table 1**, **Figure 1**).

**Table 1:** Comparison of demographic information, clinical features, and hematological parameters between NSSI and non-NSSI groups.

Variables	Total sample (n = 162)	NSSI (N = 93)	Non-NSSI (N = 69)	t/Z/χ^2^	*P*
Demographic information						
Sex, n (%)				12.727	**<0.001**
Males	57 (35.19)	22 (23.66)	35 (50.72)		
Females	105 (64.81)	71 (76.34)	34 (49.28)		
Age (years), mean (SD)	15.26 (1.63)	14.99 (1.63)	15.62 (1.56)	-2.489	**0.014**
BMI (kg/m^2^), mean (SD)	21.60 (5.04)	21.12 (4.14)	22.26 (6.02)	-1.434	0.154
Age at onset (years), mean (SD)	13.71 (1.94)	13.32 (1.75)	14.23 (2.07)	-3.020	**0.003**
Duration of illness (months), median (P_25_, P_75_)	12.00 (6.00, 24.00)	13.00 (6.00, 36.00)	12.00 (5.50, 24.00)	-1.488 ^a^	0.137
Antidepressants, n (%)				0.604	0.739
None	27 (16.67)	14 (15.06)	13 (18.84)		
SSRIs	116 (71.60)	67 (72.04)	49 (71.01)		
Others	19 (11.73)	12 (12.90)	7 (10.15)		
Clinical features						
HAMD-17 score, mean (SD)	20.62 (5.07)	22.23 (4.08)	18.46 (5.48)	5.012	**<0.001**
HAMA score, mean (SD)	21.14 (7.29)	23.11 (6.70)	18.49 (7.26)	4.182	**<0.001**
PSS score, mean (SD)	25.90 (5.66)	27.01 (4.89)	24.41 (6.29)	2.965	0.003
IAT score, mean (SD)	52.19 (15.39)	54.53 (16.11)	49.03 (13.87)	2.277	**0.024**
Hematological parameters						
Total bilirubin (umol/L), mean (SD)	11.67 (5.32)	10.65 (4.54)	13.03 (5.99)	-2.881	**0.005**
Direct bilirubin (umol/L), mean (SD)	4.49 (2.10)	4.17 (1.81)	4.93 (2.39)	-2.292	**0.023**
Indirect bilirubin (umol/L), mean (SD)	7.17 (3.39)	6.48 (2.92)	8.11 (3.76)	-3.101	**0.002**
Uric acid (umol/L), mean (SD)	349.67 (85.91)	332.23 (82.37)	373.17 (85.55)	-3.078	0.002
T3 (ng/ml), mean (SD)	1.07 (0.27)	1.04 (0.21)	1.10 (0.33)	-1.514	0.132
T4 (μg/L), mean (SD)	68.93 (14.01)	67.92 (13.20)	70.29 (15.02)	-1.065	0.289
TSH (uIU/ml), mean (SD)	1.54 (0.78)	1.69 (0.77)	1.34 (0.76)	2.893	**0.004**

NSSI, Non-suicidal Self-injury; BMI, Body Mass Index; SSRIs, Selective Serotonin Reuptake Inhibitors; HAMD-17, 17-item Hamilton Depression Rating Scale; HAMA, Hamilton Anxiety Rating Scale; PSS, Perceived Stress Scale; IAT, Internet Addiction Test; T3, Triiodothyronine; T4, Thyroxine; TSH, Thyroid-Stimulating Hormone; ^a^, Mann-Whitney U test; SD, Standard Deviation; Bolded *P* value: < 0.05.

### 3.2 Logistic stepwise regression analysis

The logistic stepwise regression analysis identified females (OR = 2.246, 95% CI = 1.032-4.888, *P* = 0.041), higher HAMD-17 score (OR = 1.183, 95% CI = 1.088-1.286, *P* < 0.001), lower indirect bilirubin (OR = 0.890, 95% CI = 0.797-0.995, *P* = 0.040), and elevated TSH levels (OR = 2.060, 95% CI = 1.254-3.385, *P* = 0.004) as independent predictors of NSSI in adolescents with MDD (**Table 2**).

**Table 2:** Independent factors associated with NSSI.

Variables	B	SE	Wald χ^2^	OR	95% CI	*P*
Sex						
Males	Ref	–	–	–	–	–
Females	0.809	0.397	4.164	2.246	1.032 - 4.888	**0.041**
HAMD-17 score	0.168	0.043	15.415	1.183	1.088 - 1.286	**<0.001**
Indirect bilirubin	-0.116	0.056	4.229	0.890	0.797 - 0.995	**0.040**
TSH	0.723	0.253	8.145	2.060	1.254 - 3.385	**0.004**

NSSI, Non-suicidal Self-injury; HAMD-17, 17-item Hamilton Depression Rating Scale; TSH, Thyroid-Stimulating Hormone; SE, standard error; OR, odds ratio; CI, confidence interval; Bolded *P* value: < 0.05.

### 3.3 ROC curve analysis

**Figure 2:**
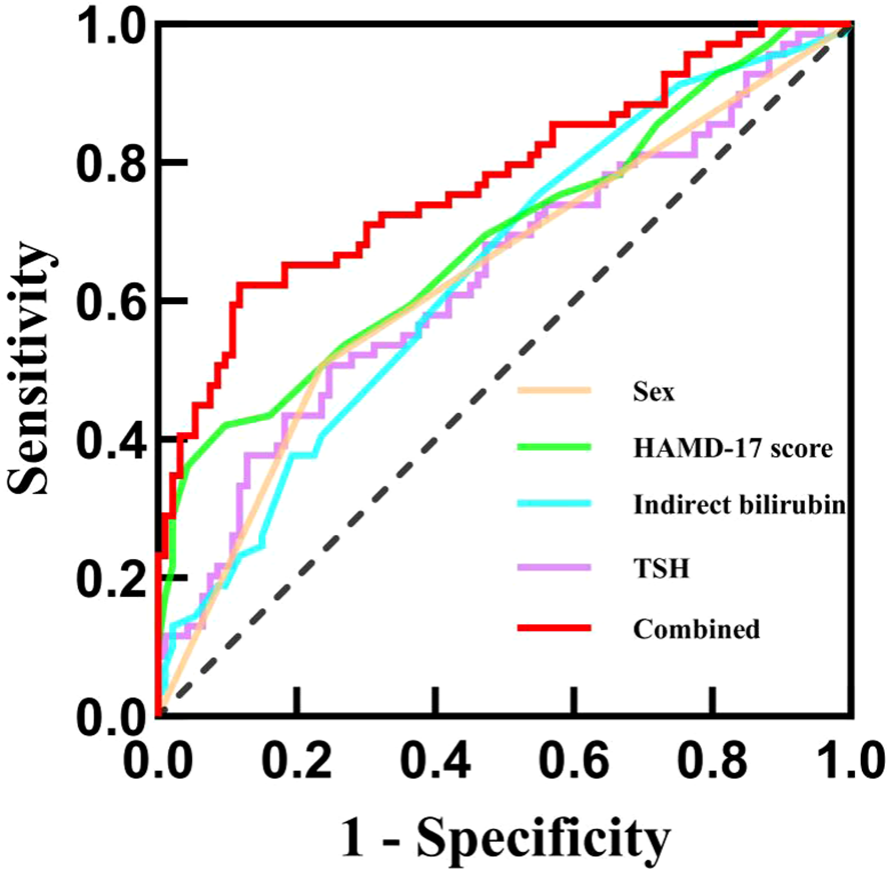
ROC curves illustrating the predictive performance of each independent risk factor for NSSI.

The predictive efficacy of each independent factor was detailed in **Table 3** and **Figure 2**. ROC curve analysis yielded the following AUC values: sex (AUC = 0.635, 95% CI = 0.548-0.723, *P* = 0.003), HAMD-17 score (AUC = 0.689, 95% CI = 0.605-0.773, *P* < 0.001), indirect bilirubin (AUC = 0.641, 95% CI = 0.556-0.727, *P* = 0.002), and TSH (AUC = 0.636, 95% CI = 0.548-0.724, *P* = 0.003). Notably, a combined multi-marker model incorporating sex, HAMD-17 score, indirect bilirubin, and TSH demonstrated a significantly enhanced discriminatory power, with an AUC of 0.776 (95% CI: 0.701-0.850, *P* < 0.001). This integrated model surpassed the performance of any single marker and exhibited robust sensitivity and specificity, indicating its superior utility in identifying adolescents with MDD at risk for NSSI.

**Table 3:** Predictive value of each independent risk factor for NSSI.

Variables	Sensitivity	Specificity	AUC	95% CI	*P*
Sex	0.763	0.507	0.635	0.548 - 0.723	**0.003**
HAMD-17 score	0.903	0.420	0.689	0.605 - 0.773	**<0.001**
Indirect bilirubin	0.452	0.754	0.641	0.556 - 0.727	**0.002**
TSH	0.753	0.507	0.636	0.548 - 0.724	**0.003**
Combined ^a^	0.882	0.623	0.776	0.701 - 0.850	**<0.001**

NSSI, Non-suicidal Self-injury; HAMD-17, 17-item Hamilton Depression Rating Scale; TSH, Thyroid-Stimulating Hormone; ^a^, Combination of sex, HAMD-17 score, indirect bilirubin and TSH; AUC, Area Under the Curve; CI, Confidence Interval; Bolded *P* value: < 0.05.

## 4 Discussion

In the present study, the detection rate of NSSI in adolescents with MDD was 57.4%, a figure that aligns closely with numerous prior cross-sectional surveys (36, 37). For instance, a survey conducted by Ren et al. among adolescents with MDD revealed that approximately 55.1% of the participants exhibited NSSI behaviors (37). Collectively, this high prevalence underscores that NSSI is no longer merely an incidental comorbidity of MDD but rather a core clinical manifestation within this population, necessitating its integration into routine clinical assessments and risk stratification systems. Furthermore, our data reveal a significantly higher proportion of NSSI among female patients compared to males. Multiple surveys focusing on adolescents with MDD have similarly identified a significantly elevated risk of NSSI in female patients (4, 38, 39). Such pronounced gender disparities likely stem from a confluence of factors. Firstly, females during adolescence often experience more intense emotional lability and internalizing symptoms, including rumination and body dissatisfaction, which may predispose them to employ self-injury as a mechanism for emotion regulation or alleviating dissociative states (40). Second, sociocultural norms may encourage females to express distress through internalized aggression, whereas males are more prone to externalizing behaviors, such as substance abuse or impulsivity (40, 41). Given these observations, clinical practice should prioritize NSSI screening for female patients with MDD and expedite the implementation of personalized interventions targeting these specific risk profiles.

At the level of clinical symptoms, univariate analysis initially revealed significant associations between NSSI and depression, anxiety, perceived stress, and IA, suggesting a multifaceted preliminary risk context for self-injurious behaviors. However, following multivariate regression analysis, only the severity of depression emerged as an independent risk factor for NSSI in adolescents with MDD. This finding implies that depressive symptom severity may serve as the core pathological mechanism driving NSSI, whereas the influences of anxiety, stress, and IA likely operate indirectly by exacerbating depressive symptoms. Possible explanations for this phenomenon are as follows. On one hand, severe depression in adolescents is frequently accompanied by heightened impulsivity, emotional expression deficits, and dysregulated emotion processing, all of which predispose individuals to NSSI (37, 42, 43). On the other hand, a neuroimaging meta-analysis demonstrated a strong positive association between depression severity and abnormal functional connectivity in the limbic system and frontoparietal network (44). Concurrently, structural or functional abnormalities in these specific brain regions have been shown to significantly increase both the frequency and severity of NSSI behaviors (45, 46). Consequently, in clinical interventions, directly targeting depressive symptoms may be paramount for mitigating NSSI behaviors in adolescents.

Regarding antioxidant markers, our study revealed that adolescents with MDD exhibiting NSSI had significantly lower bilirubin levels (particularly indirect bilirubin) compared to those without NSSI. This finding suggested a potential pathophysiological link between aberrant bilirubin metabolism and the occurrence of NSSI in this population. To our knowledge, this was one of the few studies to directly investigate the relationship between peripheral bilirubin levels and self-injurious behaviors in adolescents with MDD, offering a novel perspective on the biological mechanisms underlying NSSI. At physiological concentrations, bilirubin, especially indirect bilirubin, serves as a potent endogenous antioxidant (17). The reduced levels observed in our study may suggest signs of impaired antioxidant defense capacity in adolescents suffering from NSSI. We speculate that when endogenous antioxidant defenses are insufficient, the accumulation of ROS might compromise the functional integrity of the prefrontal-limbic circuitry, which plays a pivotal role in emotion regulation and impulse control (47). Consequently, low bilirubin levels may be indirectly associated with an increased risk of adopting NSSI as a maladaptive coping strategy, by exacerbating oxidative stress. Furthermore, bilirubin also possesses neuroprotective properties, capable of inhibiting neuronal apoptosis and promoting the expression of neurotrophic factors (48). Adolescence is a critical window for brain structural and functional remodeling, characterized by heightened sensitivity to psychosocial stressors (49). Lower bilirubin levels may diminish the brain’s buffering capacity against stress-induced damage, rendering individuals more susceptible to emotional dysregulation and prone to utilizing NSSI to alleviate psychological distress when facing interpersonal conflicts or academic pressures. Current research in this domain remains limited. Future basic studies are warranted to elucidate the specific mechanisms by which bilirubin contributes to the pathophysiology of NSSI. Moreover, it is essential to explore the feasibility of mitigating NSSI behaviors through antioxidant interventions or lifestyle modifications aimed at elevating bilirubin levels.

In terms of thyroid function, our study further identified elevated TSH levels as a critical risk factor for NSSI in adolescents with MDD. Notably, an integrated model combining TSH, indirect bilirubin, and clinical symptoms demonstrated significant predictive potential for NSSI behaviors, offering a novel biological framework for multidimensional early warning systems and personalized interventions. Previous studies have also corroborated the association between TSH and NSSI. For instance, a cross-sectional study of Chinese adolescents with MDD reported significantly higher TSH levels in patients comorbid with NSSI (24). The underlying pathophysiological mechanisms remain not fully elucidated but may involve multiple pathways. First, thyroid dysfunction may potentially exacerbate emotional instability and impulse control deficits by dysregulating serotonergic and noradrenergic systems, thereby possibly increasing the risk of NSSI (50, 51). Second, elevated TSH levels can aggravate anxiety, depression, and psychotic symptoms, subsequently precipitating NSSI behaviors (52, 53). Furthermore, abnormalities in thyroid parameters such as TSH may potentially affect brain regions critical for emotion regulation and cognitive control; dysfunction in these areas is speculated to constitute one of the key pathological bases for NSSI development (54, 55). However, consensus has not yet been reached in the literature. A retrospective study of 110 adolescents with MDD found that only elevated free thyroxine (FT4), rather than TSH, was significantly associated with NSSI (25). Conversely, another study focusing on male adolescents with MDD indicated that lower TSH levels were associated with a higher incidence of NSSI (56). This heterogeneity likely stems from differences in sample size, gender composition, disease stage, and the dynamic nature of thyroid function. In light of these discrepancies, future large-scale, multicenter, prospective longitudinal studies are warranted to elucidate the specific mechanistic roles of thyroid function indicators in adolescents with MDD comorbid with NSSI and to evaluate their utility as clinical biomarkers.

The findings of this study hold significant potential clinical implications, although they warrant cautious interpretation. First, the significant associations between bilirubin and thyroid indices and NSSI suggest that these biochemical markers could serve as auxiliary biological markers to help clinicians identify high-risk subgroups for NSSI among adolescents with MDD. Second, the links between oxidative stress, thyroid function, and NSSI shed light on underlying pathophysiological mechanisms. This suggests that, in addition to conventional psychotherapeutic interventions, future research should explore whether improving metabolic status, including regulating oxidative balance and thyroid function through lifestyle modifications or specific adjunctive therapies, could enhance the prevention of NSSI. For instance, a clinical study involving 18 adolescents with NSSI demonstrated that oral N-acetylcysteine (NAC), an antioxidant, improved clinical symptoms (57). However, until clear causal pathways and efficacy evidence are established, it is not recommended to routinely use specific medications targeting these pathways as first-line treatments for NSSI in clinical practice.

Several limitations warrant mention. First, the cross-sectional design precludes the inference of causal relationships between NSSI and the identified risk biomarkers. Although abnormalities in bilirubin and thyroid indicators may serve as potential risk factors predisposing individuals to NSSI, we cannot exclude the possibility of reverse causality. Specifically, NSSI behavior itself, as an intense psychophysiological event, may lead to secondary alterations in the levels of these biochemical markers by inducing acute stress responses, causing tissue damage, and triggering systemic inflammation. Second, the study was conducted at a single medical institution in Chaohu, Anhui, China, with a relatively small sample size, which may limit the generalizability of our findings. Third, concurrent antidepressant use in some participants may have acted as a confounding variable affecting biochemical markers. Fourth, this study was unable to fully exclude the influence of other potential confounding factors (such as nutritional and metabolic status, sex hormone levels, etc.) on NSSI. For instance, a previous cross-sectional study conducted in Spain indicated that testosterone levels were significantly associated with both the frequency and functional types of NSSI among adolescents (58). Therefore, future studies should incorporate a broader range of covariates to better control for confounding effects. Finally, although this study focused on antioxidant indicators, it did not include other key markers of oxidative stress, such as malondialdehyde (MDA) and 8-hydroxy-2’-deoxyguanosine (8-OHdG). Relying solely on single-dimensional indicators is insufficient to comprehensively reflect the body’s overall oxidative-antioxidant balance. In light of these limitations, there is an urgent need for large-scale, multicenter longitudinal studies that strictly control for medication use and multidimensional confounders, while employing more comprehensive panels of biomarkers, to further validate and extend the findings of the present study.

## 5 Conclusion

Adolescents with MDD face an elevated risk of NSSI, with females showing significantly higher susceptibility than males. Key determinants of NSSI include depression severity, antioxidant markers (specifically low indirect bilirubin), and thyroid parameters (notably elevated TSH). Consequently, clinical practice should prioritize routine screening and comprehensive assessment of NSSI in adolescents with MDD. Moreover, early intervention strategies are essential for those identified with significant risk factors.

## Data Availability

The raw data supporting the conclusions of this article will be made available by the authors, without undue reservation.

## References

[1] Lu B Lin L Su X . Global burden of depression or depressive symptoms in children and adolescents: a systematic review and meta-analysis. J Affect Disord. (2024) 354:553–62. doi: 10.1016/j.jad.2024.03.074. PMID: 38490591

[2] Wilkinson P . Non-suicidal self-injury. Eur Child Adolesc Psychiatry. (2013) 22:S75–9. doi: 10.1007/s00787-012-0365-7. PMID: 23202887

[3] Xiao Q Song X Huang L Hou D Huang X . Global prevalence and characteristics of non-suicidal self-injury between 2010 and 2021 among a non-clinical sample of adolescents: a meta-analysis. Front Psychiatry. (2022) 13:912441. doi: 10.3389/fpsyt.2022.912441. PMID: 36032224 PMC9399519

[4] Zhu Y Ren W Yang L Yue Y Lu X Zhu F . Relationship between facial emotion recognition and non-suicidal self injury in adolescents with depression: a multicenter cross-sectional study from China. J Affect Disord. (2025) 383:394–400. doi: 10.1016/j.jad.2025.05.017. PMID: 40334850

[5] Wilkinson P Kelvin R Roberts C Dubicka B Goodyer I . Clinical and psychosocial predictors of suicide attempts and nonsuicidal self-injury in the Adolescent Depression Antidepressants and Psychotherapy Trial (ADAPT). Am J Psychiatry. (2011) 168:495–501. doi: 10.1176/appi.ajp.2010.10050718. PMID: 21285141

[6] Guan M Liu J Li X Cai M Bi J Zhou P . The impact of depressive and anxious symptoms on non-suicidal self-injury behavior in adolescents: a network analysis. BMC Psychiatry. (2024) 24:229. doi: 10.1186/s12888-024-05599-1. PMID: 38532354 PMC10967160

[7] Klimes-Dougan B Begnel E Almy B Thai M Schreiner MW Cullen KR . Hypothalamic-pituitary-adrenal axis dysregulation in depressed adolescents with non-suicidal self-injury. Psychoneuroendocrinology. (2018) 102:216–224. doi: 10.1016/j.psyneuen.2018.11.004. PMID: 30590339

[8] Auerbach RP Pagliaccio D Allison GO Alqueza KL Alonso MF . Neural correlates associated with suicide and nonsuicidal self-injury in youth. Biol Psychiatry. (2020) 89:119–133. doi: 10.1016/j.biopsych.2020.06.002. PMID: 32782140 PMC7726029

[9] Kaess M Hille M Parzer P Maser-Gluth C Resch F Brunner R . Alterations in the neuroendocrinological stress response to acute psychosocial stress in adolescents engaging in nonsuicidal self-injury. Psychoneuroendocrinology. (2011) 37:157–161. doi: 10.1016/j.psyneuen.2011.05.009. PMID: 21676550

[10] Ling Y Gu Y Solomon OM Li L Chen X Wang Y . A review of the scope of non-suicidal self-injury behavior in adolescents with depressive disorders: an analysis of related influencing factors. BMC Psychiatry. (2025) 25:913. doi: 10.1186/s12888-025-07361-7. PMID: 41034762 PMC12487588

[11] Wei C Dong X . The impact of internet addiction on non-suicidal self-injury among adolescents: a moderated chain mediation model. Front Psychol. (2026) 16:1735137. doi: 10.3389/fpsyg.2025.1735137. PMID: 41613832 PMC12847421

[12] Calvete E Ayala A Jiménez-Granado A Orue I . Bidirectional associations between cyberbullying victimization, non-suicidal self-injury, and depressive symptoms in adolescents. J Adolescence. (2025) 98:119–130. doi: 10.1002/jad.70045. PMID: 40851306 PMC12780655

[13] Li M Liu F Han X Wang J Li N . The association between internet addiction and non-suicidal self-injury among adolescents: a meta-analysis. J Adolescence. (2025) 97:1433–1448. doi: 10.1002/jad.12510. PMID: 40295842

[14] Bai Y Liu W Zhang F Zheng Y Guo Q Hu H . Dysregulation of peripheral oxidative stress and the Nrf2 antioxidant system in major depressive disorder. J Affect Disord. (2025) 382:336–345. doi: 10.1016/j.jad.2025.04.066. PMID: 40258421

[15] Jiménez-Fernández S Gurpegui M Díaz-Atienza F Pérez-Costillas L Gerstenberg M Correll CU . Oxidative stress and antioxidant parameters in patients with major depressive disorder compared to healthy controls before and after antidepressant treatment: results from a meta-analysis. J Clin Psychiatry. (2015) 76:1658–1667. doi: 10.4088/JCP.14r09179 26579881

[16] Bhatt S Nagappa AN Patil CR . Role of oxidative stress in depression. Drug Discov Today. (2020) 25:1270–6. doi: 10.1016/j.drudis.2020.05.001. PMID: 32404275

[17] Zhang Y Luan H Song P . Bilirubin metabolism and its application in disease prevention: mechanisms and research advances. Inflammation Res. (2025) 74:81. doi: 10.1007/s00011-025-02049-w. PMID: 40413269

[18] Zhang C Li Y Wang W Jiang Z Liu C Kong Y . Gender differences of antioxidant system and thyroid function in depressed adolescents with non-suicidal self-injury. Neuropsychiatr Dis Treat. (2024) 20:1309–1319. doi: 10.2147/ndt.s452643. PMID: 38933097 PMC11199165

[19] Bartoli F Trotta G Crocamo C Malerba MR Clerici M Carrà G . Antioxidant uric acid in treated and untreated subjects with major depressive disorder: a meta-analysis and meta-regression. Eur Arch Psychiatry Clin Neurosci. (2017) 268:119–127. doi: 10.1007/s00406-017-0817-7. PMID: 28620773

[20] Wu Y Li J Zheng J Zhang R Liu J Xiao Y . Uric acid metabolism and brain dysfunction linking childhood maltreatment to self-harm in adolescents with mood disorders. Prog Neuro-Psychopharmacol Biol Psychiatry. (2025) 141:111460. doi: 10.1016/j.pnpbp.2025.111460. PMID: 40721167

[21] Bunevicius R . Thyroid disorders in mental patients. Curr Opin Psychiatry. (2009) 22:391–5. doi: 10.1097/yco.0b013e328329e1ae. PMID: 19387348

[22] Sawicka-Gutaj N Zawalna N Gut P Ruchała M . Relationship between thyroid hormones and central nervous system metabolism in physiological and pathological conditions. Pharmacol Reports: Pr. (2022) 74:847–858. doi: 10.1007/s43440-022-00377-w. PMID: 35771431

[23] Wu J Wang Z Xu H Yang L Liu J Zheng Y . Thyroid dysfunction in young, first-episode and drug-naïve patients with major depressive disorder: prevalence and associated clinical factors. Front Psychiatry. (2023) 14:1156481. doi: 10.3389/fpsyt.2023.1156481. PMID: 37457778 PMC10348838

[24] He Y Wei Y Wang Y Liang F Ma T . A cross-sectional study of non-suicidal self-injury in adolescent depression: association with demographic characteristics and physiological indicators. Front Psychiatry. (2024) 15:1359400. doi: 10.3389/fpsyt.2024.1359400. PMID: 39119074 PMC11306130

[25] Zhan D Jin T Zhao H Zhu H Fu J Zhang X . Hypothalamic-pituitary-thyroid axis dysregulation in adolescents with major depressive disorder and non-suicidal self-injury: a retrospective study. J Affect Disord. (2026) 399:121120. doi: 10.1016/j.jad.2025.121120. PMID: 41485512

[26] Hamilton M . A rating scale for depression. J Neurol Neurosur Psychiatry. (1960) 23:56–62. doi: 10.1136/jnnp.23.1.56. PMID: 14399272 PMC495331

[27] Zheng Y Yuan S Zhang J Ma Y He H . The sleep symptoms are directly associated with suicide risk in adolescents and youth patients with depression. Depression Anxiety. (2026) 2026:2231053. doi: 10.1155/da/2231053. PMID: 41768592 PMC12947251

[28] Li X Jin W Han L Chen X Li L . Comparison and application of depression screening tools for adolescents: scale selection and clinical practice. Child Adolesc Psychiatry Ment Health. (2025) 19:53. doi: 10.1186/s13034-025-00908-2. PMID: 40346636 PMC12065149

[29] Hamilton M . The assessment of anxiety states by rating. Br J Med Psychol. (1959) 32:50–5. doi: 10.1111/j.2044-8341.1959.tb00467.x. PMID: 13638508

[30] Jin Y Jin X Ge Z Zhang J Ding Y Wang P . The association between urinary antibiotics levels and the risk of adolescent depression. Sci Rep. (2025) 15:24093. doi: 10.1038/s41598-025-09687-4. PMID: 40619506 PMC12230150

[31] Qin Q Li J Guo Y He K . Efficacy of iTBS in adolescent depression: effects on anhedonia and exploratory moderation analyses. J Affect Disord. (2026) 403:121398. doi: 10.1016/j.jad.2026.121398. PMID: 41690635

[32] Cohen S Kamarck T Mermelstein R . A global measure of perceived stress. J Health Soc Behav. (1983) 24:385–96. doi: 10.2307/2136404 6668417

[33] Liu X Zhao Y Li J Dai J Wang X Wang S . Factor structure of the 10-item perceived stress scale and measurement invariance across genders among Chinese adolescents. Front Psychol. (2020) 11:537. doi: 10.3389/fpsyg.2020.00537. PMID: 32328009 PMC7160845

[34] Young KS . Internet addiction: the emergence of a new clinical disorder. Cyberpsychol Behav. (2009) 1:237–44. doi: 10.1089/cpb.1998.1.237. PMID: 36698420

[35] Lai CM Mak KK Watanabe H Ang RP Pang JS Ho RC . Psychometric properties of the internet addiction test in Chinese adolescents. J Pediatr Psychol. (2013) 38:794–807. doi: 10.1093/jpepsy/jst022. PMID: 23671059

[36] Li G Wang R Fu T Tong S Tian F . Depressive symptoms and subjective cognitive dysfunction associated with non-suicidal self-injury among Chinese adolescents: the mediating role of impulsivity and the moderating role of addictive features. Front Psychiatry. (2026) 17:1552165. doi: 10.3389/fpsyt.2026.1552165. PMID: 41726825 PMC12920590

[37] Ren T Wen Y Ma L Qiao D Li G Li H . Psychosocial factors affect the occurrence of nonsuicidal self-injury in adolescents with major depressive disorder through chain mediation. Eur Arch Psychiatry Clin Neurosci. (2024) 275:1209–1220. doi: 10.1007/s00406-024-01858-0. PMID: 38976048 PMC12148972

[38] Sun T Liu J Wang H Yang BX Liu Z Liu J . Risk prediction model for non-suicidal self-injury in Chinese adolescents with major depressive disorder based on machine learning. Neuropsychiatr Dis Treat. (2024) 20:1539–1551. doi: 10.2147/ndt.s460021. PMID: 39139655 PMC11319100

[39] Lv G Ma B Qi S An H . Sex differences in the prevalence and risk factors of non-suicidal self-injury behaviors among adolescent outpatients with major depressive disorder. Front Psychiatry. (2025) 16:1599627. doi: 10.3389/fpsyt.2025.1599627. PMID: 41334081 PMC12665668

[40] Chaplin TM Aldao A . Gender differences in emotion expression in children: a meta-analytic review. psychol Bull. (2012) 139:735–65. doi: 10.1037/a0030737. PMID: 23231534 PMC3597769

[41] Shen Y Hu Y Zhou Y Fan X . Non-suicidal self-injury function: prevalence in adolescents with depression and its associations with non-suicidal self-injury severity, duration and suicide. Front Psychiatry. (2023) 14:1188327. doi: 10.3389/fpsyt.2023.1188327. PMID: 37333917 PMC10272341

[42] Chen XC Xu JJ Yin XT Qiu YF Yang R Wang ZY . Mediating role of anxiety and impulsivity in the association between child maltreatment and lifetime non-suicidal self-injury with and without suicidal self-injury. J Affect Disord. (2023) 347:57–65. doi: 10.1016/j.jad.2023.11.080. PMID: 37995923

[43] Greene D Boyes M Hasking P . The associations between alexithymia and both non-suicidal self-injury and risky drinking: a systematic review and meta-analysis. J Affect Disord. (2019) 260:140–66. doi: 10.1016/j.jad.2019.08.088. PMID: 31494366

[44] Zhang Z Zhang Y Wang H Lei M Jiang Y Xiong D . Resting-state network alterations in depression: a comprehensive meta-analysis of functional connectivity. Psychol Med. (2025) 55:e63. doi: 10.1017/s0033291725000303. PMID: 40008424 PMC12080655

[45] Wu B Zhang H Chen J Chen J Liu Z Cheng Y . Potential mechanisms of non-suicidal self-injury (NSSI) in major depressive disorder: a systematic review. Gen Psychiatry. (2023) 36:e100946. doi: 10.1136/gpsych-2022-100946. PMID: 37655114 PMC10465892

[46] Zhang J Wu D Wang H Yu Y Zhao Y Zheng H . Large-scale functional network connectivity alterations in adolescents with major depression and non-suicidal self-injury. Behav Brain Res. (2025) 482:115443. doi: 10.1016/j.bbr.2025.115443. PMID: 39855474

[47] Hassamal S . Chronic stress, neuroinflammation, and depression: an overview of pathophysiological mechanisms and emerging anti-inflammatories. Front Psychiatry. (2023) 14:1130989. doi: 10.3389/fpsyt.2023.1130989. PMID: 37252156 PMC10213648

[48] Hung SY Liou HC Fu WM . The mechanism of heme oxygenase-1 action involved in the enhancement of neurotrophic factor expression. Neuropharmacology. (2009) 58:321–9. doi: 10.1016/j.neuropharm.2009.11.003. PMID: 19925812

[49] Pfeifer JH Allen NB . Puberty initiates cascading relationships between neurodevelopmental, social, and internalizing processes across adolescence. Biol Psychiatry. (2020) 89:99–108. doi: 10.1016/j.biopsych.2020.09.002. PMID: 33334434 PMC8494463

[50] Mann JJ Currier D . A review of prospective studies of biologic predictors of suicidal behavior in mood disorders. Arch Suicide Res. (2007) 11:3–16. doi: 10.1080/13811110600993124. PMID: 17178639

[51] Bauer M Heinz A Whybrow PC . Thyroid hormones, serotonin and mood: of synergy and significance in the adult brain. Mol Psychiatry. (2002) 7:140–56. doi: 10.1038/sj.mp.4000963. PMID: 11840307

[52] Liu A Yin H Feng X Sun L Liu J Zhang X . Prevalence and related factors of comorbid suicide attempts and psychotic symptoms in first-episode drug-naïve patients with major depressive disorder. BMC Psychiatry. (2025) 25:733. doi: 10.1186/s12888-025-07186-4. PMID: 40730953 PMC12309062

[53] Huang X Zhang XY . Development and validation of a prediction model for co-occurring moderate-to-severe anxiety symptoms in first-episode and drug naïve patients with major depressive disorder. Depression Anxiety. (2024) 2024:9950256. doi: 10.1155/da/9950256. PMID: 40226721 PMC11922193

[54] Bauer M Goetz T Glenn T Whybrow PC . The thyroid-brain interaction in thyroid disorders and mood disorders. J Neuroendocrinol. (2008) 20:1101–1114. doi: 10.1111/j.1365-2826.2008.01774.x. PMID: 18673409

[55] Shu Y Zhang Q Zheng Z Hou Y . Altered neural activity in adolescent major depressive disorder with nonsuicidal self-injury: a resting-state functional magnetic resonance imaging meta-analysis. Neural Plast. (2025) 2025:7885279. doi: 10.1155/np/7885279. PMID: 41281193 PMC12638161

[56] Ma J Zhao M Niu G Wang Z Jiang S Liu Z . Relationship between thyroid hormone and sex hormone levels and non-suicidal self-injury in male adolescents with depression. Front Psychiatry. (2022) 13:1071563. doi: 10.3389/fpsyt.2022.1071563. PMID: 36620661 PMC9810634

[57] Cullen KR Schreiner MW Klimes-Dougan B Eberly LE LaRiviere LL Lim KO . Neural correlates of clinical improvement in response to N-acetylcysteine in adolescents with non-suicidal self-injury. Prog Neuro-Psychopharmacol Biol Psychiatry. (2019) 99:109778. doi: 10.1016/j.pnpbp.2019.109778. PMID: 31682891 PMC7058485

[58] Calvete E Prieto-Fildalgo A Faura-García J Orue I . The role of testosterone and cortisol levels in nonsuicidal selfinjury in adolescents. J Adolescence. (2024) 96:1793–1804. doi: 10.1002/jad.12380. PMID: 39021249 PMC11618702

